# Effects of asfotase alfa on fracture healing of adult patient with hypophosphatasia and literature review

**DOI:** 10.1186/s13023-025-03663-x

**Published:** 2025-04-06

**Authors:** Songqi Wang, Lei Sun, Jing Hu, Qian Zhang, Ou Wang, Yan Jiang, Weibo Xia, Xiaoping Xing, Mei Li

**Affiliations:** Department of Endocrinology, National Health Commission Key Laboratory of Endocrinology, Peking Union Medical College Hospital, Chinese Academy of Medical Sciences and Peking Union Medical College, Beijing, 100730 China

**Keywords:** Hypophosphatasia, Adult, Asfotase alfa, Fracture healing

## Abstract

**Objective:**

Hypophosphatasia (HPP) is a rare inherited disorder caused by *ALPL* gene mutations, with fracture nonunion being a serious complication. This study investigated the effects of teriparatide and asfotase alfa (AA) on femoral fracture healing of an adult patient with HPP, accompanied with a literature review.

**Methods:**

A 37-year-old woman wheelchair-bound was diagnosed with HPP due to an extremely low serum alkaline phosphatase (ALP) level (4–10 U/L), who suffered from bilateral femur pain and non-union of femoral shaft fractures on both sides. Compound heterozygous missense mutations (c.382G > A and c.461C > T) were identified in exon5 of *ALPL* gene. The patient received teriparatide sequential AA therapy. Serum levels of ALP, β-isomerized carboxy-telopeptide of type I collagen (β-CTX) and procollagen type 1 amino-terminal peptide (P1NP), bone mineral density (BMD) and skeletal X-ray were measured during the treatment. Literature was searched by keywords of “Hypophosphatasia”, “HPP”, “ALPL”, “TNSALP”, “ALP” combined with “Asfotase alfa”, “AA”, “enzyme replacement therapy”, and “ERT”.

**Results:**

After unsuccessful 6-month teriparatide treatment for femoral fracture, AA treatment was initiated, at a dose of 2 mg/kg, 3 times a week. After the first month of AA treatment, serum ALP level increased from 4 to 9206 U/L, and serum calcium and phosphate levels decreased, with increase in PTH, β-CTX, and P1NP levels. After 4 months of AA treatment, her bone pain significantly alleviated, accompanied by significant shortening of the fracture line. After 10 months of AA therapy, the fracture demonstrated complete healing and the patient could walk independently. BMD at lumbar spine and hips was significantly increased. Among 295 adult patients with HPP reported in the literature, 213 (72.2%) exhibited skeletal-related symptoms and 91 (30.8%) presented with bone fractures. In addition to skeletal manifestations, the patients presented with early tooth loss, muscle weakness and ectopic calcification. AA treatment, spanning 9 weeks to 3 years, has been shown to increase ALP levels, promote fracture healing, improve mobility, and alleviate bone pain.

**Conclusion:**

Adult HPP patients mainly present with recurrent or poorly healing fractures, bone pain, and early loss of teeth. AA replacement therapy can effectively promote fracture healing, relieve bone pain, and enhance mobility.

## Introduction

Hypophosphatasia (HPP) is a rare inherited metabolic bone disorder that is caused by pathogenic mutations in the *ALPL* gene, which follows autosomal dominant or recessive inheritance patterns [1]. The *ALPL* gene, located on chromosome 1, consists of 12 exons and encodes the tissue-nonspecific isoenzyme of alkaline phosphatase (TNSALP) [2, 3]. TNSALP is predominant in skeleton, liver, kidney, and teeth [4]. Hypophosphatasia is characterized by diminished enzymatic activity of TNSALP, which results in accumulation of its substrates in bone, including pyridoxal 5′-phosphate (PLP), phosphoethanolamine (PEA), and inorganic pyrophosphate (PPi) [5]. PPi impairs bone mineralization and leads to deposition of the pyrophosphate crystals in joints. The estimated prevalence of severe and moderate HPP in European populations is approximately 1 in 300,000 and 1 in 6370, respectively [6, 7].

According to clinical characteristics and the age of onset, HPP is usually categorized into six forms: perinatal, benign prenatal, infantile, childhood, adult, and odonto HPP [8, 9]. Adult patients with HPP typically occur in middle age and commonly present with pain in weight-bearing bones, stress fractures, muscle weakness, early loss of permanent teeth, impaired ambulation [1, 10]. Overall fracture incidence is higher in adult HPP patients than general population over 18 years old, and fractures are often difficult to heal [11, 12], of which nearly three-quarters of the patients require surgery, notably, implant surgeries frequently fail in adult patients with HPP, leading to mobility impairments and psychological problems [13, 14].

HPP used to lack effective treatment and recently enzyme replacement therapy significantly relieves patients' clinical symptoms [15–17]. Asfotase alfa (AA), a recombinant human TNSALP, can improve the symptoms of children with HPP, but there is less research about its application in adult patients with HPP [18, 19]. This study prospectively observed the effects of teriparatide and sequential AA treatment on bone fractures healing in an adult HPP patient and conducted a comprehensive analysis of the therapeutic effect of AA in adult patients with HPP through reviewing all studies on adult patients with HPP receiving AA treatment.

## Materials and methods

### Object

The patient, a 37-year-old woman, was admitted to the Department of Endocrinology of Peking Union Medical College Hospital (PUMCH) in September 2022 due to "bilateral lower limb pain for four years". She was born at full term with a birth weight of 3.75 kg, and the length was unknown. She was breastfed for one year and she showed no significant differences in growth, development, intelligence or activity compared to her peers. She experienced early loss of her permanent teeth (the exact age was forgotten) and underwent full-mouth dental implant surgery at the age of 24 years. Since 2018, she has experienced progressively worsening lower limb pain, along with decreased tolerance for activity and a diminished quality of life. By the end of 2022, she was unable to walk independently and relied on assistance for daily activities. She denied that her parents were consanguineously married and reported no family history of similar diseases. Physical examination revealed that her height was 162 cm, weight was 44.0 kg, and BMI was 16.77 kg/m^2^. She entered the clinic room in a wheelchair and had all 28 teeth as dental implants. The thyroid was not enlarged, and there were no obvious abnormalities in the lungs or heart. Both hands displayed ulnar deviation of the middle fingers. No other significant skeletal deformities were noted, although mild atrophy of the lower limb muscles was observed.

The study protocol was approved by the scientific ethics committee of PUMCH. The patient signed informed consent before participating in this study.

### Laboratory evaluation

Fasting blood samples were collected in the morning at about 8:00 AM. Serum calcium, phosphate, ALP, alanine aminotransferase (ALT), and creatinine (Cr) were analyzed using an automatic biochemical analyzer (ADVIA 1800, Siemens, Germany). An automated Roche electrochemiluminescence system (Roche Diagnostics, Switzerland) was used to detect serum concentrations of parathyroid hormone (PTH), and 25 hydroxyvitamin D (25-OHD), β-isomerized C-terminal telopeptide of type I collagen (β-CTX), and procollagen type 1 amino-terminal peptide (P1NP). In addition, erythrocyte sedimentation rate (ESR) was measured using an automatic biochemical analyzer (ADVIA 1800, Siemens, Germany). Free triiodothyronine (FT3), free thyroxine (FT4), thyroid-stimulating hormone (TSH), blood cortisol, and carcinoembryonic antigen (CEA) were measured using an automated Roche electrochemiluminescence system (Roche Diagnostics, Switzerland). Serum immunofixation electrophoresis (IFE) was performed using an electrophoresis instrument (ProteomeLab, Beckman Coulter, United States) for differential purposes. All biochemical indicators were measured uniformly in the central laboratory of PUMCH.

### Bone mineral density and X-ray examination

Dual-energy X-ray absorptiometry (DXA, Lunar Prodigy, GE Healthcare, Madison, WI, USA) was used to measure areal bone mineral density (BMD) at lumbar spine 1–4 (LS), femoral neck (FN), trochanter, and total hip (TH) at baseline and follow-up. Radiographs of the spine, pelvis, and bilateral lower limbs were obtained at baseline. Based on the patient's clinical presentation and radiological findings, regular follow-up radiographs of both femora were scheduled every 3–6 months to closely monitor the healing process of the fractures.

### Detection of gene mutation

Genomic DNA was extracted from the peripheral leukocytes of the patient using the QIAamp DNA Mini Kit (Qiagen, Germany) according to the manufacturer's instructions. Mutation detection was performed using next-generation targeted exon capture sequencing. All mutations and potential pathogenic variants were validated by Sanger sequencing. The pathogenicity of the variants was assessed according to the American College of Medical Genetics and Genomics and Association for Molecular Pathology (ACMG/AMP) Standards and Guidelines. The pathogenicity of the missense mutation was predicted using MutationTaster software (http://www.mutationtaster.org/).

### Treatment and follow-up

After being diagnosed with adult-HPP complicated by non-healing bilateral fractures, severe bone pain, and limited mobility, the patient requested further treatment. As AA was not yet available in mainland China, the patient was treated with experimental subcutaneous daily injections of 20 µg teriparatide to promote fracture healing. Simultaneously, the patient received daily supplementation with 600 mg calcium and 0.25 µg calcitriol every other day. However, after 6 months of the above treatment, bone pain persisted, and there was no radiographic evidence of healing in the bilateral femoral fractures.

The patient then initiated AA (Strensiq, Alexion Pharmaceuticals Inc., Boston, MA, USA) therapy and discontinued teriparatide treatment. Based on previous studies, we recommended administration of AA treatment for the patient through subcutaneous injections of 2 mg/kg per dose, three times a week [12, 20]. At 1, 4, 6, and 10 months of AA treatment, bone metabolic markers, BMD, femoral fracture imaging, and drug-related adverse reactions were monitored. The AA dosage was adjusted according to the improvement in the patient’s symptoms and fracture healing status. Initially, the patient received 2 mg/kg per dose, three times a week for 4 months, followed by 1 mg/kg per dose, three times a week for 2 months, and finally, 1 mg/kg per dose, once a week for 4 months.

### Literature review

We reviewed relevant studies from the following medical databases: PubMed, Web of Science, Embase, and the Cochrane Central Register of Controlled Trials (CENTRAL) up to October 2024 to identify case reports, case series, and research articles written in English on AA treatment for adult patients with HPP. The keywords used in the literature search included “Hypophosphatasia”, “HPP”, “*ALPL*”, “TNSALP”, and “ALP” in combination with “Asfotase alfa”, “AA”, “enzyme replacement therapy”, “ERT”. These keywords were combined using the Boolean operators. Relevant studies were identified by screening article titles and abstracts. The inclusion criteria were studies on AA treatment in adult patients with HPP. Exclusion criteria included studies on AA treatment in adolescents, conference abstracts, and articles for which the full text was unavailable.

### Statistical analyses of the data from the literature

The Kolmogorov–Smirnov test was performed to assess the normality of continuous variables. Categorical data, such as gender, form of HPP, symptoms, gene classification, and symptom improvement after AA therapy, were expressed as frequencies and percentages. Continuous variables with normal distribution, such as age, serum phosphorus, and serum calcium, were presented as mean ± SD, while those with non-normal distribution, such as serum ALP, were presented as median (interquartile range, IQR). The Mann–Whitney U test was used to compare changes in serum ALP, PLP, phosphorus, calcium, PTH, β-CTX, and P1NP before and after AA treatment. A *P* value of less than 0.05 was considered statistically significant. Statistical analyses were performed using SPSS software (version 25.0; IBM Corp., Armonk, NY, USA).

## Results

### Baseline characteristics of the patient in this study

The patient was a young woman who experienced early loss of both primary and permanent teeth, along with progressively worsening bilateral thigh pain over time. Laboratory tests revealed extremely low serum ALP levels (4-10U/L) (reference range: 35–100 U/L), elevated serum phosphorus levels of 1.56 mmol/L (reference range: 0.81–1.45 mmol/L), and low 25-OHD levels of 17.3 ng/mL (reference value: > 30 ng/mL). Other bone metabolic indicators included serum calcium of 2.47 mmol/L (reference range: 2.13–2.70 mmol/L), β-CTX of 0.28 ng/mL (reference range: 0.21–0.44 ng/mL), P1NP of 27.2 ng/mL (reference range: 15.1–58.6 ng/mL), and PTH of 29.5 pg/mL (reference range: 15–65 pg/mL). Serum levels of FT3, FT4, TSH, ALT, Cr, blood cortisol, CEA, IFE were all within normal limits. X-ray examination revealed an incomplete fracture of the bilateral femoral shafts without evidence of healing.

### Variant of the ALPL gene

**Fig. 1:**
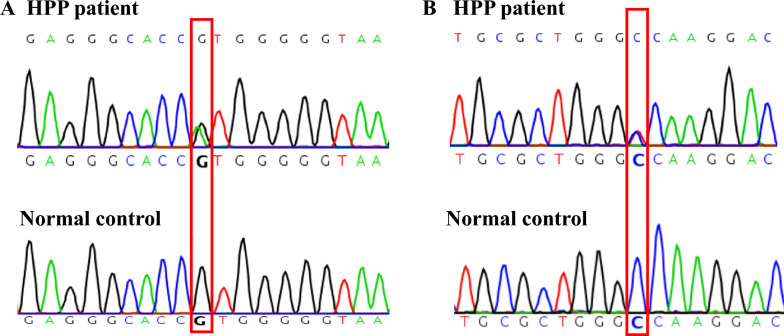
ALPL mutation in this patient revealed by whole-exome sequenc. **A** ALPL exon 5 c.382G > A heterozygous variant. **B** ALPL exon 5 c.461C > T heterozygous variant

Genetic testing showed compound heterozygous missense mutations (c.382G > A and c.461C > T) in exon 5 of *ALPL* gene (Fig. 1). According to the ACMG/AMP Standards and Guidelines, both mutations were pathogenic (the former: PM2 + PM3 + PP3 + PP4 + PS3; the latter: PM2 + PP3 + PP4 + PM3). The Mutation Taster software also predicted that both mutations were pathogenic.

### Effects of AA treatment on patient with HPP in this study

#### Changes in clinical symptoms

After 1 month of AA treatment, the patient reported relief from bone pain, allowing her to stand and walk slowly. After 4 months of treatment, bone pain was significantly relieved, and mobility was notably improved. After 6 months of AA treatment, her bone pain completely disappeared and and she could walk 2 km independently. By 10 months of AA treatment, the patient resumed normal daily life and work.

#### Changes in serum biochemical indicators

After 1 month of AA therapy, serum ALP concentration significantly increased to 9206 U/L, up from 4 U/L. Accompanied by an increase in bone metabolism markers, including β-CTX (0.53–0.79 ng/mL), P1NP (83.9–75.9 ng/mL), and PTH (17–49.3 pg/mL), as well as a decrease in serum calcium (2.46–2.21 mmol/L) and phosphorus (1.73–1.45 mmol/L).

**Fig. 2:**
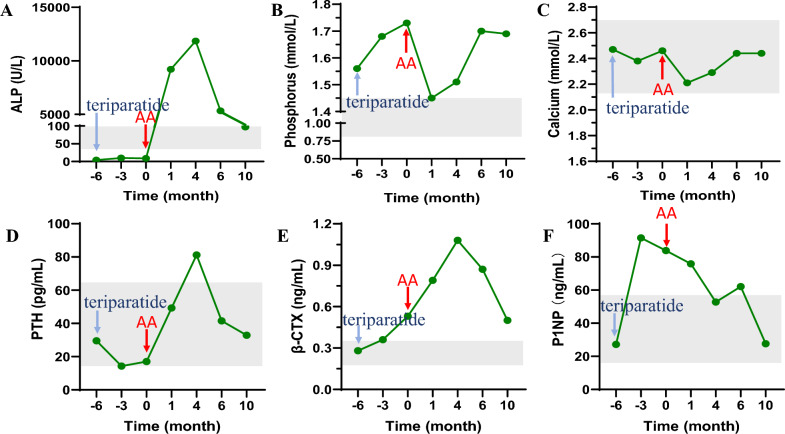
Changes of bone metabolic markers during teriparatide and sequential AA therapy. **A** Serum levels of ALP during the follow-up. **B** Serum levels of phosphorus during the follow-up. **C** Serum levels of calcium during the follow-up. **D** Serum levels of PTH during the follow-up. **E** Serum levels of β-CTX during the follow-up. **F** Serum levels of PINP during the follow-up. Annotation: The blue arrow indicates the start of teriparatide treatment. The red arrow indicates the start of AA treatment. The gray area represents the normal reference range. *AA* asfotase alfa, *ALP* alkaline phosphatase, *PTH* parathyroid hormone, *β-CTX* β-isomerized carboxy-telopeptide of type I collagen, P1NP = procollagen type 1 amino-terminal peptide

After 4 months of AA treatment, the dosage was reduced to 1 mg/kg per dose, three times per week, due to significant improvement in the patient's clinical symptoms and fracture healing. After 2 months, the ALP level had decreased by a half (ALP at 5332 U/L). At the same time, serum level of β-CTX decreased while serum calcium and phosphate levels increased. Accordingly, PTH levels also decreased. The AA dosage was then further reduced to 1 mg/kg per dose, once a week. After 4 months, the ALP level remained at the upper limit of the normal range (ALP at 97 U/L). β-CTX decreased but remained above the upper limit of the normal range, P1NP normalized, serum calcium and phosphate levels stabilized, and PTH levels continued to decrease. All laboratory data at baseline and during treatment are presented in Table 1 and Fig. 2.

**Table 1:** Changes of bone turnover biomarkers and BMD during teriparatide and sequential AA therapy

	Baseline	Teriparatide for 3 mons	Teriparatide for 6 mons	AA for 1 mon	AA for 4 mons	AA for 6 mons	AA for 10 mons	Reference range
Ca (mmol/L)	2.47	2.38	2.46	2.21	2.29	2.44	2.44	2.13–2.70
P (mmol/L)	1.56	1.68	1.73	1.45	1.51	1.70	1.69	0.81–1.45
PTH (pg/mL)	29.5	14.3	17	49.3	81.2	41.5	32.9	15.0–65.0
25-OHD (ng/mL)	17.3	20.5	19.4	17.4	17.8	21.6	13.2	30–100
ALP (U/L)	4	10	9	9206	11,843	5332	97	35–100
β-CTX (ng/mL)	0.28	0.36	0.53	0.79	1.08	0.87	0.50	0.21–0.44
P1NP (ng/mL)	27.2	91.5	83.9	75.9	52.8	62.1	27.6	15.1–58.6
ALT (U/L)	20	21	18	14	23	18	16	7–40
AST (U/L)	22	26	20	19	25	21	16	13–35
Alb (g/L)	46	43	43	43	42	45	46	35–52
Cr (umol/L)	56	52	53	55	57	55	56	45–84
LS aBMD/Z score	1/029/− 0.2	–	–	–	–	–	1.165/1.0	–
FN aBMD/Z score	0.788/− 0.6	–	–	–	–	–	0.774/− 0.8	–
TH aBMD/Z score	0.741/− 1.4	–	–	–	–	–	0.800/− 1.0	–

*AA* asfotase alfa, *mon* month, *Ca* total calcium, *P* phosphorus, *PTH* parathyroid hormone, *25-OHD* 25-hydroxyvitamin D, *ALP* alkaline phosphatase, *β-CTX* β-isomerized carboxy-telopeptide of type I collagen, *P1NP* type 1 N-terminal propeptide, *ALT* alanine aminotransferase, *AST* aspartate aminotransferase, *Alb* albumin, *C* creatinine, *LS* lumbar spine, *aBMD* areal bone mineral density, *FN* femur neck, *TH* total hip

#### Changes in BMD and fracture imaging

At baseline, BMD at the LS and TH were 1.029 g/cm^2^ and 0.741 g/cm^2^, with Z-scores of -0.2 and -1.4, respectively. After 6 months of teriparatide treatment followed by 6 months of sequential AA therapy, BMD at LS and TH improved to 1.165 g/cm^2^ and 0.800 g/cm^2^, with Z-scores of 1.0 and -1.0 (Table 1).

**Fig. 3:**
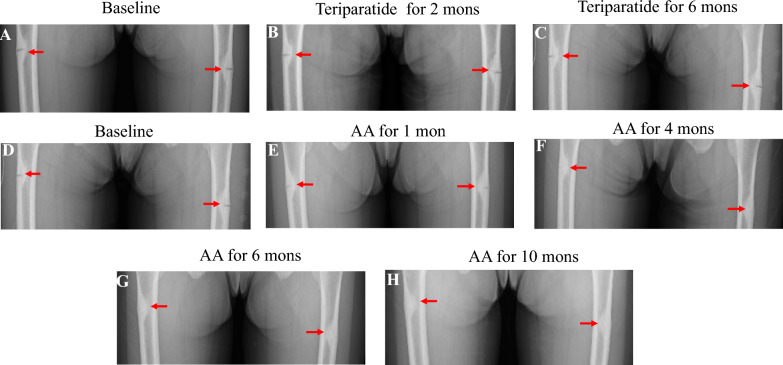
Changes of bilateral femoral fractures during teriparatide treatment, and sequential AA therapy. Annotation: The arrows in the above figure indicate the bilateral proximal lateral femoral fractures. *AA* asfotase alfa

After 4 months of AA treatment, the fracture line in both femurs were shortened compared to baseline. After 6 months of AA treatment, bilateral femur fractures were nearly healed. Complete fracture healing was achieved with no visible radiolucency after 10 months of AA treatment (Fig. 3).

#### Safety during AA treatment

The patient reported a local injection site reaction, including redness and localized itching, within one day of AA injection. Except for that, she did not report symptoms of blurred vision, decreased vision, visual field defects, painful urination, hematuria, or other related discomforts. Throughout the treatment course, a decrease in the patient's calcium levels was observed following medication, but it remained above the lower limit. Additionally, liver enzyme and Cr levels remained consistently within the normal range.

#### Results of the literature review

**Fig. 4:**
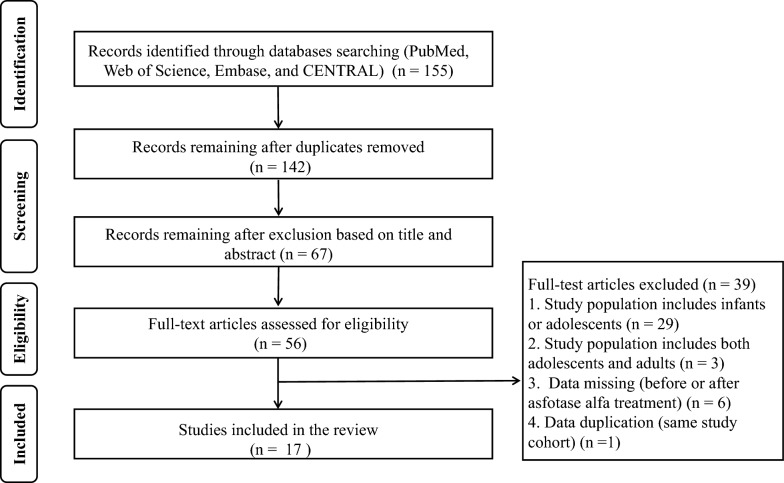
Flow diagram of study selection process. *CENTRAL* The Cochrane Central Register of Controlled Trials

As shown in Fig. 4, a total of 155 references were identified from the databases. Among them, the treatment effects of AA in adult patients with HPP were evaluated in 17 studies, which comprised 12 case reports (n = 21) and 5 clinical studies (n = 274) (Tables 2 and 3) [13, 14, 18, 20–33].

**Table 2:** Summaries of characteristic of adult patients with hypophosphatasia by literature review

	Adult patients with hypophosphatasia (N = 295)
Age (mean ± SD), year	45.14 ± 16.68
Female, % (n)	65.4% (193)
Male, % (n)	34.6% (102)
Form of HPP, % (n)	
Pediatric-onset	91.5% (270)
Adult-onset	8.5% (25)
Symptom, % (n)	
Bony pain	79.7% (235)
Fractures history	30.8% (91)
Muscle weakness	49.4% (146)
Dental	71.9% (212)
Neurologic	17.6% (52)
Gene classification, % (n)	
Heterozygous	92.2% (272)
Homozygous	2.7% (8)
Unknown/missing	5.1% (15)

**Table 3:** Summaries of adult patients with hypophosphatasia before and after receiving AA treatment by literature review

	n	Before AA treatment	After AA treatment	*P* value
Serum alkaline phosphatase (U/L)	75	11.57 ± 6.35	5873.00 ± 2787.62	0.001
Serum P-pyridoxal-5-phosphate (mmol/L)	26	545.00 ± 87.47	13.87 ± 5.95	0.008
Serum phosphorus (mmol/L)	27	1.68 ± 0.18	1.36 ± 0.07	0.024
Serum calcium (mmol/L)	27	2.39 ± 0.09	2.33 ± 0.09	0.038
parathyroid hormone (pg/ml)	26	30.60 ± 8.98	51.00 ± 14.19	0.005
type 1 N-terminal propeptide	24	47.31 ± 17.45	91.29 ± 15.06	0.009
C-terminal telopeptide of type l collagen (ng/mL)	24	0.37 ± 0.09	0.72 ± 0.24	0.13
Fracture healing	–	–	10/11	–
Bony pain alleviated	–	–	65/78	–
Mobility enhanced	–	–	61/62	–
Quality of life improved	–	–	69/74	–

*AA* asfotase alfa

Among 295 adult patients with HPP, the mean age was 45.14 ± 16.68 years, with 193 women and 102 men. 91.5% (270/295) had pediatric-onset HPP, and only 8.5% were classified as adult-onset HPP. The majority of reported cases receiving AA treatment were Caucasian, with only four patients being Japanese. No reports or studies have documented Chinese patients receiving AA treatment, as AA has not been approved for marketing in China. Among these patients with HPP, 30.8% (91/295) had a history of bone fractures. 49.4% (146/295) had muscular manifestations of HPP, with some patients experiencing limited daily activities. 71.9% (212/295) had dental manifestations, 79.7% (235/295) had a history of pain, and 17.6% (52/295) had neurological abnormalities.

Among the 17 studies, 76.5% (13/17) used a treatment dosage of 6 mg/kg per week for AA, with some administering it as three injections per week and others as six. After AA therapy, 83.3% of patients reported relief from bone pain, 98.4% experienced improved mobility, and 93.2% reported enhanced quality of life. However, the literature review identified only a few case reports that explored the effectiveness of AA therapy in fracture healing for adult patients with HPP. Among them, 10 patients showed improvement in fracture healing. The average healing time after AA treatment was 14.25 ± 6.68 months, with the shortest reported healing time being 6 months and the longest 24 months.

After AA treatment, all studies reported a significant increase in serum ALP activity, with a median level of 5873.00 ± 2787.62 U/L and a highest recorded level of 13,336 U/L. Additionally, the levels of PTH, osteocalcin, P1NP, and β-CTX were increased, while serum calcium and phosphate levels decreased.

Regarding safety evaluation, 28.1% (77 of 274) of patients reported injection site reactions (ISRs) during treatment, while 20.8% (57 of 274) developed injection site lipodystrophy resembling lipohypertrophy. Three patients experienced severe allergic reactions, primarily manifesting as difficulty breathing, choking sensation, swelling of the eyes, lips, or tongue, dizziness, vomiting, fever, and chills, which required discontinuation of AA therapy. The patient in this study reported local injection reactions within one day of AA administration, including skin redness and localized itching, with no signs of lipodystrophy, hypersensitivity reactions, or abnormalities in liver and kidney function.

## Discussion

We diagnosed with and treated a female adult patient with HPP who had suffered from bone pain, bilateral femoral fractures, and loss of mobility. Six months of teriparatide treatment failed to relieve her bone pain or promote fracture healing. After 10 months of AA treatment, she achieved fracture healing and regained the ability to walk. As AA has not yet been approved in China, this study presents the first report on the effectiveness of AA treatment in adult patients with HPP in the country, providing valuable clinical experience in AA replacement therapy for this patient group. Through a literature review, we found that adult patients with HPP typically present with bone hypomineralization, severe bone pain, recurrent and non-healing fractures, and poor dental health. Laboratory tests showed extremely low ALP levels and elevated serum calcium and phosphate levels. AA treatment can effectively increase serum ALP levels, reduce TNSALP enzyme substrates, promote fracture healing, relieve bone pain, enhance mobility, and improve the quality of life in adult patients with HPP.

HPP is a genetic bone disease caused by the *ALPL* gene mutations, which encodes TNSALP [34, 35]. TNSALP is expressed in multiple tissues and plays a critical role in hydrolyzing PPi, which facilitates bone mineralization through the formation of hydroxyapatite when combined with calcium [36]. When mutations occur in the *ALPL* gene, TNSALP activity is reduced, resulting in the accumulation of its substrates, including PPi, PLP, and PEA [10, 37]. PPi inhibits bone mineralization by osteoblasts and chondrocytes, and its accumulation disrupts the mineralization of hydroxyapatite by impairing calcium and phosphate integration, leading to osteoid accumulation, a hallmark of rickets and osteomalacia [14, 38].

AA is the first pharmacological treatment for HPP by bone-targeted enzyme-replacement therapy [29]. AA is a soluble human recombinant TNSALP fusion protein (726 amino acids, homodimer) comprising the catalytic domain of human TNSALP, the human IgG Fc domain, and a deca-aspartate bone-targeting peptide [39, 40]. It is approved for paediatric-onset HPP and can rapidly improve bone mineralization, pulmonary function, motor function, cognitive development, catch-up height-gain, muscle strength and daily activity ability [41–44]. In adult HPP patients, AA effectively reduces TNSALP substrate levels and markedly improves motor function and health-related quality of life (HRQoL) [45]. AA treatment can also improve fracture healing, and enhance walking ability and reduce bone pain [23].

AA is generally well-tolerated, with most adverse events being mild to moderate in severity. The most frequent adverse reactions are injection site reactions, including erythema, discoloration, pain, pruritus, swelling, induration, macule, bruising, and nodules among others [46]. In addition, most patients exhibites lipodystrophy at the injection site, primarily characterized by faint initial signs of soft tissue distension during the first 3 months of treatment, including bulging of subcutaneous fat tissue suggesting lipohypertrophy. Upon palpation, no bulky fat masses were identified, instead, there was sagging of the skin, indicating dystrophy of the subcutaneous fat tissue. As long-term adverse effects, patients may experience ectopic calcification in the eyes and kidneys. However, it is currently believed that this ectopic calcification may also result from the HPP disease itself.

A second-generation TNSALP enzyme replacement therapy, efzimfotase alfa, has been developed for the treatment of HPP. It hydrolyzes PPi at approximately twice the rate of AA and supports once-weekly injections. A phase 1 study demonstrated that efzimfotase alfa showed acceptable safety, tolerability, and PK profiles, and resulted in dose-dependent reductions in plasma levels of TNSALP substrates, including PPi and PLP, in adults with HPP [47]. This may be a safe and effective treatment option for HPP patients in the future.

HPP is a heritable disorder caused by pathogenic *ALPL* variants, with over 450 ALPL variants and 850 genotypes reported. Severe HPP forms often result from homozygosity or compound heterozygosity, moderate forms from missense variant dominant negative effects, and milder forms from haploinsufficiency [48, 49]. With advances in genome-editing technology, a study using transcription activator-like effector nucleases (TALENs) corrected the c.1559delT mutation, resulting in the recovery of ALP activity in vitro [50]. Another study showed that a single injection of an adeno‐associated virus vector serotype 8 harboring TNSALP‐D_10_ effectively improved the long bone phenotype in adult HPP mice [51]. These preclinical studies suggested that gene editing may have important prospects for HPP treatment.

We prospectively observed the effects of AA on bone pain and fracture nonunion for the first time in adult patients with HPP. AA treatment significantly improved the healing of bilateral fractures, relieved bone pain, and enhanced the patient's mobility. However, there were some limitations to this study. First, First, we did not measure the concentrations of TNSALP substrates, such as PLP and PPi, during AA therapy. Second, we did not quantify the physical function, pain, or quality of life in the HPP patient. Third, research in the literature on the efficacy of AA in adult HPP patients is extremely limited, which restricts our comprehensive understanding of its effects in this population. Therefore, it is necessary to conduct prospective long-term studies on a large sample of HPP patients receiving AA treatment.

## Conclusion

Nonunion of fractures is a serious complication of adult HPP patients. AA treatment can significantly promote fracture healing, alleviate bone pain and improve mobility. The second-generation enzyme replacement therapy shows promising potential. In the future, gene editing therapy is worth further research in HPP patients.

## Data Availability

The datasets used and/or analysed during the current study are available from the corresponding author on reasonable request.

## Abbreviations

HPP: Hypophosphatasia
AA: Asfotase alfa
ALP: Alkaline phosphatase
β-CTX: β-isomerized C-terminal telopeptide of type l collagen
P1NP: Procollagen type 1 amino-terminal peptide
BMD: Bone mineral density
TNSALP: The tissue nonspecific isoenzyme of alkaline phosphatase
PLP: Pyridoxal 5′-phosphate
PEA: Phosphoethanolamine
PPi: Pyrophosphate
ALT: Alanine aminotransferase
Cr: Creatinine
PTH: Parathyroid hormone
25-OHD: 25 Hydroxyvitamin D
FT3: Free triiodothyronine
FT4: Free thyroxine
TSH: Thyroid-stimulating hormone
CEA: Carcinoembryonic antigen
IFE: Immunofixation electrophoresis
DXA: Dual-energy X-ray absorptiometry
BMD: Areal bone mineral density
LS: Lumbar spine
FN: Femoral neck
TH: Total hip
CENTRAL: The Cochrane Central Register of Controlled Trials
IQR: Interquartile range
ISRs: Injection site reactions
HRQoL: Health-related quality of life
